# Airborne Prokaryote and Virus Abundance Over the Red Sea

**DOI:** 10.3389/fmicb.2019.01112

**Published:** 2019-05-31

**Authors:** Razan Z. Yahya, Jesús M. Arrieta, Michael Cusack, Carlos M. Duarte

**Affiliations:** ^1^Division of Biological and Environmental Science and Engineering, Red Sea Research Centre and Computational Bioscience Research Center, King Abdullah University of Science and Technology, Thuwal, Saudi Arabia; ^2^Spanish Institute of Oceanography (IEO), Oceanographic Center of The Canary Islands, Santa Cruz de Tenerife, Spain

**Keywords:** aerosol microparticles, bioaerosol, prokaryotic cell, viral-like particles, particulate matter, Red Sea

## Abstract

Aeolian dust exerts a considerable influence on atmospheric and oceanic conditions negatively impacting human health, particularly in arid and semi-arid regions like Saudi Arabia. Aeolian dust is often characterized by its mineral and chemical composition; however, there is a microbiological component of natural aerosols that has received comparatively little attention. Moreover, the amount of materials suspended in the atmosphere is highly variable from day to day. Thus, understanding the variability of atmospheric dust loads and suspended microbes throughout the year is essential to clarify the possible effects of dust on the Red Sea ecosystem. Here, we present the first estimates of dust and microbial loads at a coastal site on the Red Sea over a 2-year period, supplemented with measurements from dust samples collected along the Red Sea basin in offshore waters. Weekly average dust loads from a coastal site on the Red Sea ranged from 4.6 to 646.11 μg m^−3^, while the abundance of airborne prokaryotic cells and viral-like particles (VLPs) ranged from 77,967 to 1,203,792 cells m^−3^ and from 69,615 to 3,104,758 particles m^−3^, respectively. To the best of our knowledge, these are the first estimates of airborne microbial abundance in this region. The elevated concentrations of resuspended dust particles and suspended microbes found in the air indicate that airborne microbes may potentially have a large impact on human health and on the Red Sea ecosystem.

## Introduction

The atmosphere is a dynamic system that transports a variety of particles suspended in air (known as aerosols), which include a diverse range of particles of both natural and anthropogenic origin, and are emitted from marine and freshwater surfaces, plants, soils, humans, animals, industry, and agriculture etc. (Yamaguchi et al., 2012; Fiałkiewicz-Kozieł et al., 2016). In addition to inorganic and organic particles, aerosols contain microorganisms, such as viral particles, prokaryotic, and eukaryotic cells, that are transported through the atmosphere and can often carry pathogens and produce toxins that are harmful to humans, animals, and plants (Yamaguchi et al., 2012). Atmospheric processes play a significant role in the dynamic fluxes of airborne microbial abundance by governing aerosolization and deposition fluxes of dust and microbes (Mayol et al., 2014; Morris et al., 2014), where rain drops, wind speed and direction are the main contributing factors to the deposition flux of airborne microbes (Peter et al., 2014). Wind speeds must be of sufficient strength to lift microbes from the ground and into the atmosphere (Sammonds and Thompson, 2007). Once in the air, microbes can remain suspended for several days; sufficient time to be transported across thousands of kilometers before being deposited onto surfaces ( Burrows et al., 2009; Mayol et al., 2017). Indeed, Louis Pasteur first demonstrated the presence and transport of microorganisms and spores in the air, which he proposed was responsible for the spread of some infectious diseases (Tan and Rogers, 2007). Since then, the study of the release and transport of microbes into the atmosphere has grown considerably (Nguyen et al., 2006; Burrows et al., 2009; Mayol et al., 2017). Yet, the microbial component of natural aerosols has received comparatively far less attention than the mineral fraction (Griffin, 2007).

Prokaryotes and some viruses can be efficiently transported through the air over long distances as they remain suspended for long periods due to their small size (Knight, 1980). However, viruses require a host to survive during transport and, thus, are typically associated with bacteria attached to Particulate matter (PM) (Yang et al., 2011; Reche et al., 2018). Indeed, the atmosphere is a conduit for the rapid transport of microbes, prokaryotes, and viruses, both of marine and terrestrial origin, with characteristic transport distances of thousands of kilometers (Després et al., 2012; Mayol et al., 2017). Wind-driven aerosolization and resuspension processes that deliver cells from land and marine ecosystems to the atmosphere, and depositional processes that transfer airborne microbes to land and ocean, support the highly dynamic load and transport of microbes over the marine boundary layer of the atmosphere (Mayol et al., 2014, 2017). Many factors might affect the survival of prokaryotes and viruses in the air (e.g., high temperature, nutrients, grazers, desiccation, and exposure to intense UV radiation), (Mojica and Brussaard, 2014; Finke et al., 2017). Thus, it is difficult to determine how many prokaryotes and viruses reach their final destination in a viable state and, therefore, to quantify the real impact of atmospheric microbial transport on the receiving ecosystems (Hijnen et al., 2006).

Desert dust is recognized as one of the main sources of airborne microorganism (Kellogg and Griffin, 2006), as the atmosphere over desert areas typically contains a high load of microbes that can be transported over large distances (Kellogg and Griffin, 2006). Dust deposition is, however, concentrated across a number of global hot spots that act as a sink for airborne microbes, which may affect the general human and ecosystem health in some seasons (Sultan et al., 2005). The Arabian Peninsula is one of the major sources of aeolian dust on the planet (Jish Prakash et al., 2015), producing intense and widespread sand storms that are generated in the Arabian Peninsula and the Saharan desert.

The Red Sea, located between these two major dust sources, is a global hot spot for dust deposition, receiving a very large input of dust, estimated at 6 TG of dust annually (Jish Prakash et al., 2015). Whereas these inputs have received attention in terms of the effects on atmospheric processes (Jish Prakash et al., 2015) and their role as a potential source of nutrients to the oligotrophic Red Sea (Chase et al., 2011), the associated loads and potential inputs of prokaryotes and viruses have not been examined.

Here we hypothesize that the high dust loads over the Red Sea, a large sink for dust on the planet, are likely to be related to high loads of airborne organisms, including prokaryotes and viruses. We tested this hypothesis through a 2-year assessment of temporal variability of the dust concentrations as well as prokaryotes and viral-like particle (VLP) loads in dust collected from a coastal location on the central Red Sea. Our results are supplemented with information from dust samples collected in research cruises along the Red Sea in offshore water. We also examine the partitioning of Prokaryotes and VLPs between particle-associated and free prokaryotic cells and free viral particles.

## Materials and Methods

### Dust Sampling Sites

Particulate matter and airborne microbes were collected for 2 years (from December 2015 to December 2017) from a coastal site on the Red Sea at KAUST (King Abdullah University of Science and Technology, Thuwal, Saudi Arabia, 22° 18′ 17.5788″ N, 39° 6′ 8.6832″ E). In addition, samples were collected offshore in the Red Sea at different locations sampled along nine different research cruises along the Saudi EEZ of the Red Sea (Supplementary Figure 1 and Supplementary Table 1).

### Sampling Collection and Preparation

Regular sampling of PM was performed using automatic sequential high-volume samplers (MCV-CAV), equipped with PM cut off inlets at a flow rate of 20 m^3^ hr^−1^ over periods of 24 h to 1 week. Air was sampled through the inlet by means of an in-built pump. The ambient air was filtered to collect the PM on acid-treated low metal glass microfiber filters (Whatman 150 mm diameter). Before sampling filters were combusted at 200°C for 24 h and pre-conditioned at ambient temperatures and relative humidity (21°C and 60% RH) prior to sampling. The mass concentration of PM was determined by weighing the filters before and after sampling on a microbalance in a climate-controlled atmosphere and expressed as μg m^−3^. Filters were weighed 3 times and the average of the three measurements are reported here. Moreover, the high-volume sampler on board the research vessel was placed on the top deck at an elevation of ∼7.5 m high over the sea level and it was equipped with a weather vane, which would switch off the pump and thus cease sampling immediately whenever the sampler was downwind of the ship’s exhaust, thereby avoiding contamination.

Samples of airborne microbes were collected weekly and prepared according to Mayol et al. (2014). The quantitative estimates of prokaryote and viral abundance were carried out using a commercially available cyclonic collector (Coriolis sampler, Bertin Technologies), located on a pier at an elevation of ∼3 m above sea level, while the sampler on board the research vessel was placed on a deck at an elevation of ∼7.5 m above sea level. Samples were taken only when the sampler was not downwind of the ship’s exhaust to avoid contamination. All elements of the sampler in contact with the sampling solution were cleaned by immersion in a 4% HCl solution for at least 12 h before sampling and washed with Milli-Q water and stored in clean Ziplock bags prior to sampling. Air was aspirated into the cone and pre-filled up to 15 ml with a collection liquid containing Milli-Q water and 0.005% Triton X-100, in a whirling motion to form a vortex at a flow of 300 L min^−1^ over 6 h (equivalent to 108 m^3^). In this system, particles are pulled against the wall and separated from the air due to centrifugal force and are thus concentrated in the liquid. Immediately after the sample was collected, 5 ml of each sample were fixed with 2% formaldehyde (final concentration) for 10 min, for subsequent counting of prokaryotic cells in the laboratory. Cells were filtered onto 0.4 μm-pore-size black polycarbonate filters and stained with DAPI (4′, 6-diamidino-2-phenylindole, 1 μg ml^−1^ final concentration). The cells were mounted onto microscope slides and stored at −20°C until analysis. Moreover, another 5 ml were fixed with glutaraldehyde (0.5% final concentration) for VLP counting. Particles were filtered through a 0.2 μm Millipore polycarbonate filter, incubated for 10 min in the dark, frozen in liquid nitrogen and stored at −80°C until analysis.

### Detachment Procedure of Bacteria and Viruses From Aerosol Particles

In order to determine the total VLPs and prokaryotic cells (i.e., including free and particle-attached), a detachment procedure was carried out according to the method of Reche et al. (2018). Samples were treated with sodium pyrophosphate (0.1% final concentration), followed by the detergent Tween 20 (0.5% final concentration), and shaking at ∼720 rpm for 30 min, followed by sonication for 1 min. The particles were then extracted by centrifugation by mixing 1 ml of Nycodenz and 1 ml of the treated aerosol sample in Eppendorf tubes. The tubes were centrifuged (Eppendorf 5415 R) at 14,000 g for 90 min at 4°C. Following centrifugation, the VLPs and prokaryotic cells were easily extracted from the distinct separated layer, which was fixed again and stored at −20°C for the prokaryotic cells slides, and at −80°C for the VLP tubes, until analysis.

### Microbial Abundance

A *Leica* DM 1000 epifluorescence microscope was used to determine the free and total prokaryotic cells abundance under 1,000 magnifications. The microscope was equipped with an HBO 50 mercury arc lamp and a filter cube containing a 360/40 BP excitation filter, a 400-nm dichromatic mirror and a 470/40 BP emission filter. Bright particles presenting strong blue fluorescence were identified as prokaryotes, as result of DAPI bound to DNA at ∼390 nm when excited with 365 nm light (Porter and Feig, 1980). At least 200 cells were counted per sample in at least eight different fields. For low abundance samples where 200 cells could not be found, such as field blanks, a minimum of 200 fields was counted in order to provide statistically significant estimates (Mayol et al., 2014). The blank was subtracted from each sample after analysis. VLPs were prepared for counting by flow cytometry following the procedure described in Gasol and Morán (2016); samples were liquefied in a cold-water immersion, diluted 50–100 fold in filtered Tris–EDTA, stained with 100x SYBR green (1 × final concentration) and incubated at 80°C in a water bath for 10 min, followed by incubation in the dark for 5 min at room temperature. A BD FACSCanto II Flow Cytometer was used to determine the quantification of the free and total VLPs. Fluorescent 1 μm beads (BD Bioscience) were used as a standard to convert microbial counts to abundance. After adding 10 μl of the10^7^ beads solution, each sample and its corresponding blank were run for 2 min at a low flow rate. In addition, a control was run before the prepared samples by using 25 μl of autoclaved and 0.2 μm Millipore polycarbonate filtered TE buffer and 5 μl of SYBR Green I. The blank and the controls were subtracted from each sample after analysis by plotting green fluorescence against side scatter, the detection of the viral population was calculated.

### Particles Loss by Re-aerosolization Accounting

Collecting airborne microbes using liquid systems, such as in our sampler, may result in sample losses by re-aerosolization of the sample. Re-aerosolized particles may be carried away by the air flowing through the system (Williams, 1982; Riemenschneider et al., 2010), leading to underestimation of the total microbial load. Thus, we calculated the loss of 1 μm-size particle using the same conditions for samples collected in the field and a similar sampling of air flow. We followed the procedure described by Mayol et al. (2014). Briefly, 1 μm diameter fluorescently labeled polystyrene microspheres (Invitrogen) were added to the sampling cup (∼250,000 beads ml^−1^). As the amount of liquid in the cup is kept constant, losses by aerosolization can be determined by the number of microspheres remaining after 1 hr in the sampler. The remaining beads abundance was determined using flow cytometry (Mayol et al., 2014). The particle number in the liquid (N ml^−1^) can be modeled as

(1) $$\frac{\mathrm{dN}}{\mathrm{dt}}=\mathrm{kN}$$

in which the initial abundance (*N*_0_) = *N* (*t* = 0).

(2) $$N(t)=N_{0}.\;e^{\mathrm{kt}}$$

Then the accumulation of prokaryotic and viral particles in the sampling cup can be expressed as

(3) $$\frac{\mathrm{dN}}{\mathrm{dt}}=-0.7701N+c$$

In which the initial particles number in the blank N(t) can be expressed as

(4) $$N(t)=1.29853c+0.999998N_{0}\cdot e^{-0.7701t}-1.29853c\cdot e^{-0.7701t}$$

where N(*t*) is the microbial abundance in the sample, and *N*_0_ is the microbial abundances in the field blank, (N ml^−1^). Moreover, the abundance of the particles ml^−1^ h^−1^ can be expressed using the formula

(5) $$c=\frac{0.999998N_{0}\cdot e^{-0.7701t}-N_{0}(t)}{1.29853\;e^{-0.7701t}-1.29853}$$

As the sampling conditions in our case are 300 l of air per 15 ml over 6 h, which is the equivalent of 1.2 m^3^ml^−1^h^−1^. Accordingly, the *C_air_* can be expressed as

(6) $$C_{\mathrm{air}}=\frac{c}{1.2}$$

where *C_air_* is the microbial abundance in the air (particles m^−3^).

Finally, the microbial abundance in the air was calculated using the particle abundance found in the blank and in the sample and the sampling time

(7) $$C_{\mathrm{air}}=\frac{0.641751N_{0}-0.641751e^{0.7701t}\cdot N(t)}{1-e^{0.7701t}}$$

where *t* is the collection time (hours).

### Deposition Flux and Trajectory Calculation

The Red Sea basin is an extremely arid desert region with negligible annual rainfall. Thus, wet deposition in the region is minimal and only dry deposition is included in our calculations. We estimated the deposition flux (F_D_) from:

$$F_{D}=V_{D}C_{p}$$

where V_*D*_ is the dry deposition velocity (cm s^−1^) and C_*p*_ is the concentration of particles/viruses or Prokaryotes in the air (μg/cells m^−3^) (Jurado et al. (2004). The deposition velocity of airborne material can be determined from the particle diameter and density, and from wind speed, temperature, and humidity (Williams, 1982). Wind speeds, which ranged from 4 to 29 ms^−1^, were averaged over the 6-h sampling period. The deposition of prokaryotes was calculated based on a density of 1.46 g cm^−3^ and 0.5 μm (Steward and Preston, 2011; Brum et al., 2013; Mayol et al., 2014). The deposition velocity of viruses was determined as a function of the deposition velocity of particles (density of 1.5 g cm^−3^ and diameter of 2 μm) in the air as viruses exist on the surface of the particles.

Air mass back trajectory analysis is a useful tool for observing the large-scale movement of air masses over time. Air masses can carry atmospheric aerosols long distances from their point of origin, and thus provide insight into the potential sources of atmospheric particulates. We calculated 72-h air mass backward trajectories, and calculated the median suspension time of bacterial cells in the atmosphere (Mayol et al., 2017) arriving at our sampling location at KAUST, for elevations of 100, 300, and 500 m above sea level. The location of our sampling site, roughly halfway down the lateral coast of the Red Sea, was affected predominantly by prevailing Northerly winds (most common), Saharan Desert or Arabian Peninsula air masses or, less frequently, air masses from the Southern Red Sea and Arabian Gulf. Air mass back trajectories were calculated using the HYSPLIT transport and dispersion model^1^.

### Data Analysis

Analysis of variance (ANOVA) and the *post hoc* Tukey HSD test were used to determine the statistical differences between seasons. We also used (R Software, R Core Team, 2018) to check the statistically significant differences between seasons.

**FIGURE 1:**
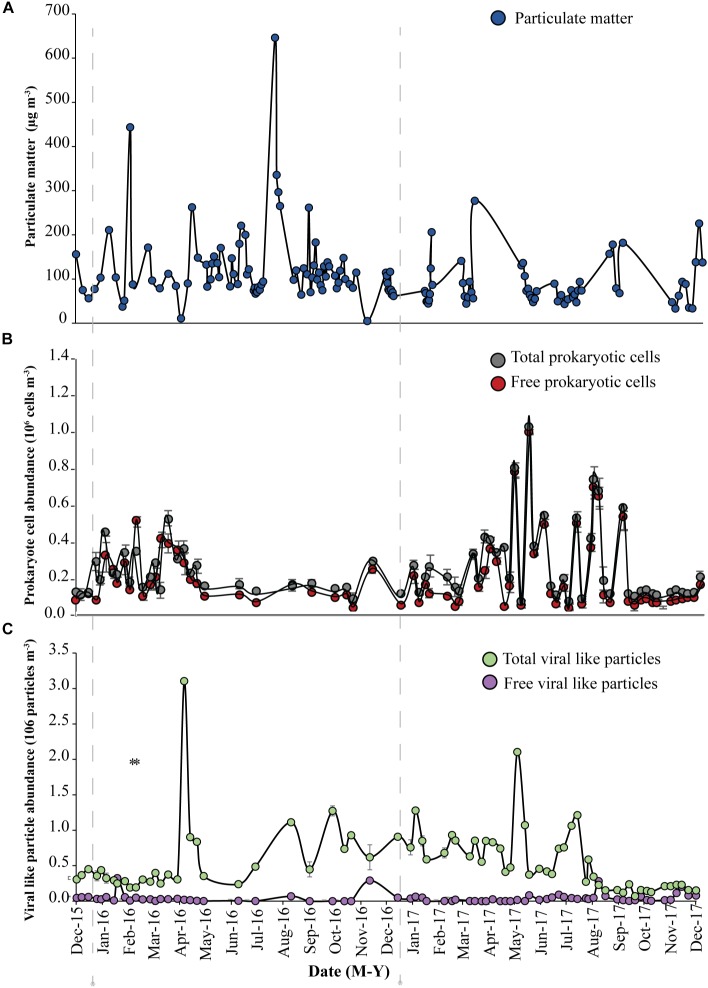
Abundance of **(A)** Particulate matter (blue circles), **(B)** total prokaryotic cells (gray circles) and free prokaryotic cells (red circles), **(C)** total viral-like particles (VLPs), (green circles) and free viral-like particles (VLPs), (purple circles) in the coastal site on the Red Sea at KAUST. The gray dash-lines indicate the beginning of samples collected in 2016 and 2017, respectively.

## Results

### Quantification of Aerosol Dust Particulates and Airborne Microbes Over the Red Sea

**FIGURE 2:**
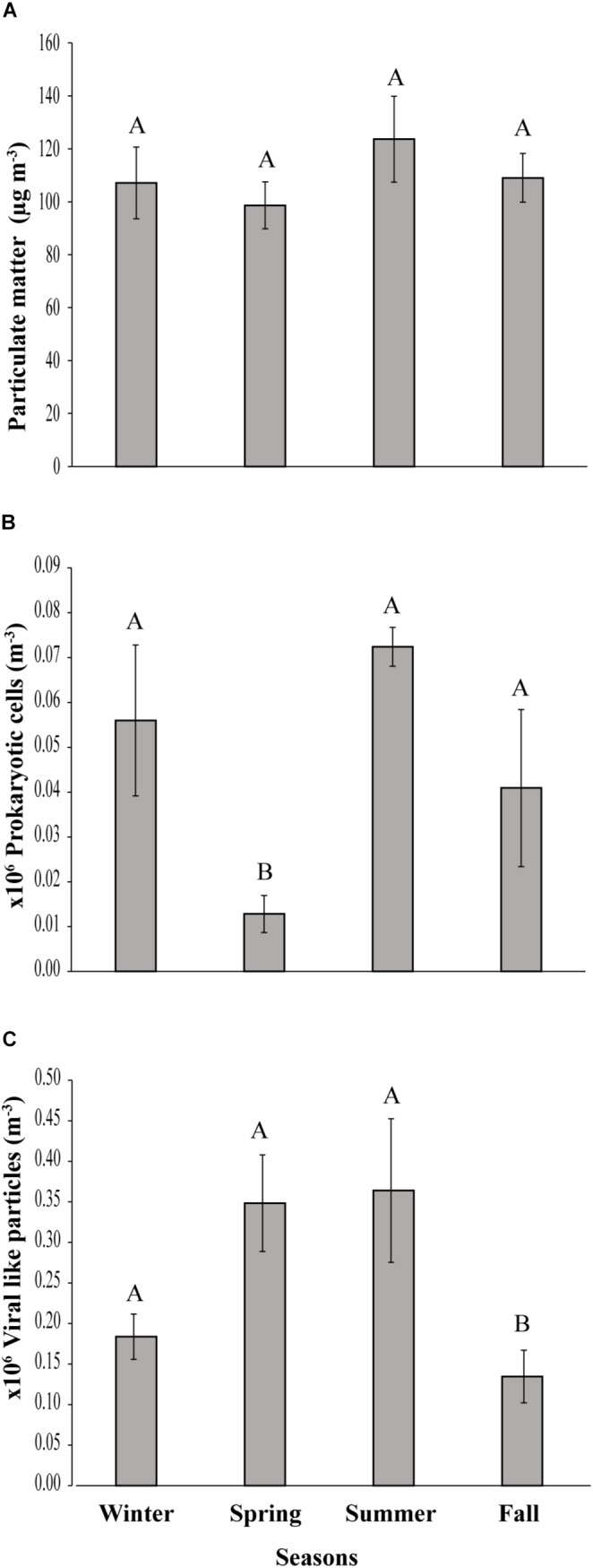
Seasonal variation of **(A)** Particulate matter, **(B)** prokaryotic cells and **(C)** viral-like particles in the coastal site on the Red Sea at KAUST. Different letters represent significant differences (ANOVA *p* < 0.05).

A load of aerosol dust at the coastal location in the central Red Sea (KAUST) ranged from 4.6 to 646 μg m^−3^, (average 110 μg m^−3^). Airborne total prokaryotic cell concentrations ranged 15-fold from 77,967 to 1,203,792 cells m^−3^ (average ± SD 292,582 ± 218,289 cells m^−3^). Around 55% of the prokaryotic cells were attached to dust particles, in which the free prokaryotic cells ranged 30-fold from 37,976 to 1,168,792 cells m^−3^ (237,385 ± 224,121 cells m^−3^). The total VLPs concentration ranged 45-fold from 69,615 to 3,104,758 particles m^−3^, (561,415 ± 476,281 particles m^−3^). Most (93%) of the VLPs were attached to dust particles, as the free VLPs load ranged between 18 and 324,215 particles m^−3^ (41,763.4 ± 63,306 viral particles m^−3^) (Figure 1 and Table 1). Hence, we measured a ratio of about 2:1 VLPs per prokaryotic cell in the airborne community (range from 0.2 to 25.2, median = 1.8). The abundance of airborne prokaryotic cells and VLPs were independent of the dust load (*P* = 0.4 and *P* = 0.9), respectively, even when high loads of dust loads and airborne microbiota were more likely to occur simultaneously in the summer when temperatures are highest (Table 2). Our prokaryotic cell data showed significant differences in spring only, whereas the VLP data showed significant differences in autumn only. PM did not present a significant difference among seasons (Figure 2), We observed a negative relationship between prokaryotic cells and VLPs abundance with wind speed (*R*^2^ = 0.03, *P* = 0.11) and (*R*^2^ = 0.02, *P* = 0.19), respectively, but we recorded a significant positive relationship between PM and wind speed (*R*^2^ = 0.04, *P* = 0.02).

The dust load in samples collected offshore along the Saudi Coast ranged from 30 to 664 μg m^−3^ (average 110 μg m^−3^). The total airborne prokaryotic cells ranged 8-fold between 43,088 to 326,489 cells m^−3^ (154,812 ± 65,255 cells m^−3^). Almost all the cells (99.9%) were attached to dust particles, as the free prokaryotic cells ranged 10-fold between 23,088 to 221,489 cells m^−3^ (118,859 ± 53,181 cells m^−3^). In addition, the total VLPs ranged 16-fold from 70,317 to 1,118,141 particles m^−3^ (358,826 ± 444,427 particles m^−3^). Around 75% of VLPs were attached to dust particles, in which the free VLPs ranged 4-fold from 28,055 to 98,841 particles m^−3^ (122,098 ± 32,843 particles m^−3^). Therefore, loads of airborne microbes in the open Red Sea were about half the airborne microbe concentration measured at the coastal station in the Red Sea, despite a similar average dust load. The lowest abundances of suspended dust particles and airborne prokaryotes and VLP abundance over the Red Sea were detected around 25 N and 26 N, respectively, with a somewhat higher VLP to prokaryote cell ratio of about 4:1(range from 0.6 to 18.3, median = 1.02), compared to that observed at the coastal station.

**Table 1:** Abundance, deposition fluxes of aerosol prokaryotic cells and viral particles in coastal and offshore sites along the Red Sea.

	Location	Abundance (particle m^−3^)	Estimated deposition flux (particle m^−2^ s^−1^)
Prokaryotic cells	Coastal	292,582 ± 218,289 (73) (77,967–1,203,792)	503 ± 373 (128 – 2033)
	Offshore	154,8 ± 65,255 (21) (43,088 – 326,489)	305 ± 122 (82 – 620)
Total VLPs	Coastal	561,415 ± 476,281 (73) (69,615 – 3,104,758)	970 ± 848 (118 – 5827)
	Offshore	358,826 ± 444,427 (5) (70,317 – 1,118,141)	760 ± 1014 (131 – 2519)

*Values shown are average ± SD and the number of observations (n), and the range (in brackets).*

**Table 2:** Coefficient of determination (R^2^) and *P* values between temperature and wind speed with PM, total Prokaryotes and total VLPs.

	Particulate matter	Total prokaryotic cells	Total viral-like particles
Temperature	*R*^2^ = 0.07 *P* = 0.02	*R*^2^ = 0.06 *P* = 0.03	*R*^2^ = 0.06 *P* = 0.03
Wind speed	*R*^2^ = 0.04 *P* = 0.02	*R*^2^ = 0.03 *P* = 0.11	*R*^2^ = 0.02 *P* = 0.19

### Deposition Flux and Transport of Dust Particles and Airborne Microbes

The calculated depositional flux of prokaryotic cells at the central Red Sea location sampled over 2 years ranged from 128 to 2033 cells m^−2^ s^−1^ (503 ± 373 cells m^−2^ s^−1^), whereas the deposition flux of total VLPs ranged from 118 to 5827 particles m^−2^ s^−1^, (970 ± 848 particles m^−2^ s^−1^). The calculated deposition flux of prokaryotic cells and VLPs in samples collected offshore along the Saudi Coast ranged from 82 to 620 cells m^−2^ s^−1^ (305 ± 122 cells m^−2^ s^−1^) and from 131 to 2519 particles m^−2^ s^−1^ (760 particles ± 1014 m^−2^ s^−1^, Table 1).

Backward air mass trajectory analysis showed that most of the PM samples collected in the Central Red Sea were transported by air masses from the northwest (36% of sampling events), followed by the north (21% of samples), and south (13% of samples), with only a minor number of samples collected under northeast and southeast air masses (10% of all samples). The lowest contributing air masses were from the west and east (3% of samples). In contrast, most of the prokaryotic cells and VLPs samples collected in the Central Red Sea were transported by air masses from the northwest (26% sampling events), followed by the north (22% of samples) and southeast (16% of samples). Samples from the south (14% of samples) and northeast (11% of samples) were limited, and there were minor contributions from the west, east, and southwest air masses (4%, 4% and 3% of samples, respectively).

**FIGURE 3:**
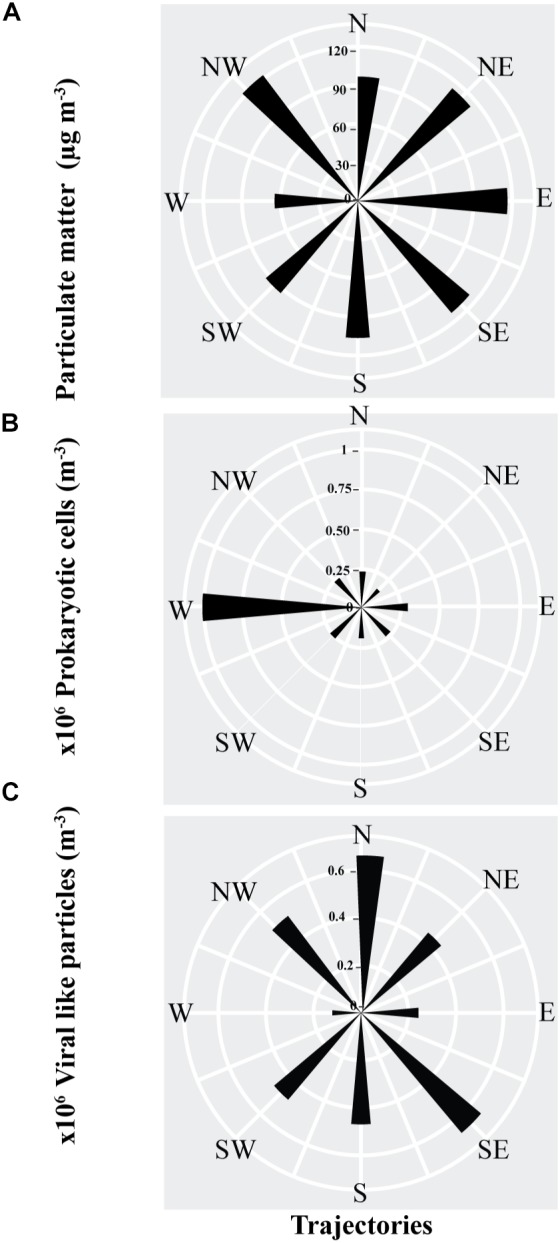
The average abundance, as represented by the length of the sectors, of **(A)** Particulate matter, **(B)** prokaryotic cells **(C)** viral-like particles in air masses sampled from different back-trajectories in the coastal site on the Red Sea at KAUST.

The PM and VLPs abundances were independent of air mass origin, with the highest loads found in air masses originating in the northwest (i.e., the Saharan Desert or the Mediterranean Sea), and from the north (the Saharan Desert or Arabian Peninsula) for the VLPs. The abundance of airborne prokaryotic cells was strongly dependent on the source of the air mass, with air masses originating in the west (i.e., Africa) supporting average prokaryote loads about 3 to 7-fold higher than air masses arriving from other regions (Figure 3).

**FIGURE 4:**
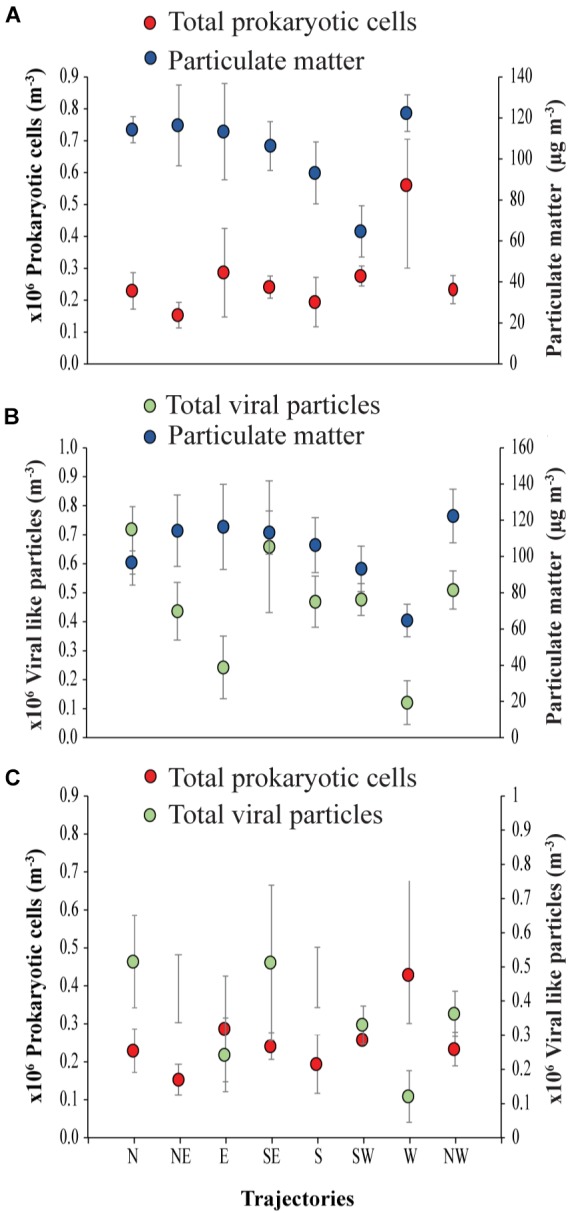
Air mass backward trajectories of **(A)** prokaryotic cells to Particulate matter, **(B)** viral particles to Particulate matter and **(C)** prokaryotic cells to viral-like particles in the coastal site on the Red Sea at KAUST.

The ratio of viral particles to dust particle showed that air masses supported loads of 1869 to 6845 viral particles per dust particle m^−3^. In contrast, there were 1340 to 9753 prokaryotic cells per dust particle m^−3^. Moreover, loads of the prokaryotic cells in all the trajectories were about 1 to 5-fold higher than the viral particles (Figure 4).

## Discussion

Our results confirm the presence of high loads of prokaryote and VLPs in dust suspended in the atmosphere over the Red Sea. Recent assessments of the chemical composition of dust in the northern Red Sea region report dust loads ranging from 20 to 30 μg m^−3^ (Torfstein et al., 2017; Torfstein and Kienast, 2018), which are 22-fold lower than those reported here. However, comparison of our results with other related studies is precluded by the absence of studies the quantifying the abundance of aerosol prokaryotes and VLPs particles in the Red Sea or even in the Mediterranean Sea, through which some of the air masses delivering microbes to the Red Sea transit. Only one study has reported deposition rates of VLP’s before, but this referred to virus deposited in mountains in southern Spain (Reche et al., 2018). Our calculations show deposition rates seven orders of magnitude lower than those reported in mountains in Spain (Reche et al., 2018). No additional studies on VLPs in aerosols are available. However, research using culture-dependent techniques and amplicon sequencing of dust-associated microbes have been reported for the Mediterranean and Northern Red Sea (Kellogg and Griffin, 2006; Griffin et al., 2007; Polymenakou et al., 2008; Raisi et al., 2010; Katra et al., 2014; Rosselli et al., 2015; Rahav et al., 2016; Federici et al., 2018). These studies report the impacts of airborne microbes and how it can play an important role in the ocean. Moreover, compared to the surface water of the Red Sea in the same location of our study, prokaryotic cells in surface seawater ranged from 1.46 to 4.80 × 10^5^ cells ml^−1^ (Silva et al., 2019) This is two to four orders of magnitude higher than the concentration of airborne prokaryotes. This confirms that atmosphere is a small reservoir for prokaryotes, compared to the large prokaryote biomass in marine ecosystems (Cotner and Biddanda, 2002; Kirchman, 2008). Indeed, the only global assessment available to date, using the same methods as applied here, reported an average of 8020 cells m^−3^ across the subtropical and tropical open ocean (Mayol et al., 2014); 37-fold lower than the average measured here for the Central Red Sea (292,582 cells m^−3^), and 19-fold lower than the offshore (154,812 cells m^−3^). The airborne prokaryote loads observed here (about 1.9 × 10^4^ bacteria m^−3^ ) are also an order of magnitude higher than those characteristically found in the atmosphere over land (Bauer et al., 2002). Some of these differences may be due to differences in methods used, as different collection methods and counting methods are available, such as qPCR (Cho and Hwang, 2011), plate counting (Lighthart and Shaffer, 1995), flow cytometry (Bowers et al., 2011b), and microscopic counting (Bowers et al., 2011a). However, the Red Sea is located in the Global Dust Belt (Prospero et al., 2002), flanked by two deserts in the Arabian Peninsula and Saharan desert, which act as important sources of dust, and render the Red Sea a major hotspots of Aeolian dust in the planet receiving very high dust inputs (Jish Prakash et al., 2015). The heavy dust loads in the atmosphere overlaying the Red Sea could explain the large microbial loads and depositional fluxes reported here in the Red Sea. However, collecting airborne microbes using liquid systems, as in our case, might result in sample losses by re-aerosolization (Riemenschneider et al., 2010). This could lead to an underestimation, not overestimation, of the prokaryote loads estimated for the Red Sea, which would, therefore, render our estimates as conservative, but comparable to those of Mayol et al. (2014), using similar methods. Mayol et al. (2014) sampled in the open ocean, away from the nearest land, and showed that airborne bacteria abundance declined with distance from the coast. This suggests that, in addition, to the high dust loads over the Red Sea, the proximity to land even in the open Red Sea also contributes to support elevated loads of airborne bacteria.

Our results characterize the atmosphere over the Red Sea as a global hot spot for airborne prokaryote concentrations. This is consistent with the very high dust loads in the Red Sea (mean 110 μg m^−3^). However, we observed that the load of airborne biota is not proportional to dust load, and may be governed by microbial abundance at the source sites, soils, or ocean, and modified subsequently by losses (mortality and deposition) of the organisms during atmospheric transport. Strong winds play an important role in the transportation of microbes far from their source by the adhesion of microbes to dust particles (Griffin, 2007), which is reflected in the positive correlations between the dust particles and wind speed and temperature. Additionally, we found that 55% and 99.9% of the prokaryotic cells in offshore and onshore water, respectively, were attached to dust particles, as previously described in many other studies, indicating that airborne bacteria are mostly attached to dust (Polymenakou et al., 2008; Després et al., 2012; Yamaguchi et al., 2012) or to organic particles (Aller et al., 2005, 2017). VLPs attached to dust particles were substantially higher with 93% and 75% in offshore and onshore water, respectively, as similarly described in Weinbauer et al. (2009).

Long-distance transport at high altitudes exposes microbes to challenging conditions, particularly very low humidity and intense UV radiation, which may act as a strong selective force, leading to transport of the best-adapted microbes only (Smith et al., 2011). The most likely survivors of long-distance transport in dust clouds are bacterial spores (Griffin, 2007; Hervas and Casamayor, 2009). Furthermore, airborne prokaryote cells appear to be associated with transparent exopolymeric particles that absorb UV wavelengths and might prevent complete dehydration (Reche et al., 2018). Thus, these particles may contribute to the persistence of prokaryotic cells and VLPs in the atmosphere through long-distance transport.

In our study, we found that the loads of airborne microbes in the open Red Sea were about half those of the coastal site, as it has been proved that the bacteria over oceans are lower compared to the bacteria over land (Prospero et al., 2005; Burrows et al., 2009). Moreover, the ratio of VLP particles to prokaryote cells was 2:1 at the coastal station in the Central Red Sea, and somewhat higher at 4:1 VLPs per prokaryotic cell in the offshore Red Sea samples. The enrichment of VLPs relative to prokaryotic cells may be due to the longer residence times of VLPs in the atmosphere, due to their small size, as reflected in their much higher calculated deposition rate compared to prokaryotes, thus allowing them to be transported much greater distances (Reche et al., 2018).

The backward trajectories of the air masses sampled here reflect the dominance of NW winds in the Central Red Sea and suggest that dust and airborne microbiota loads are relatively independent of air-mass source. The relationship between dust, prokaryote and VLPs load and temperature is consistent with the higher abundance of dust and bio-aerosols in the summer months. Indeed, seasonal changes in airborne microbial abundance and diversity have been reported previously in the Korean Peninsula (Lee et al., 2010), although the drivers for such seasonal variability remain unknown. Comparing seasonal patterns across sites is, however, cumbersome as the use of different methods to determine the bacterial abundance can affect the estimates of bacterial abundance and diversity (Fahlgren et al., 2011).

Calculated depositional fluxes over the Red Sea provided a higher VLPs count compared to prokaryotic cells, estimated with a mean of 970 VLPs m^−2^ s^−1^ and 503 prokaryotic cells ^−2^ s^−1^ in the onshore samples, and 760 VLPs m^−2^ s^−1^ and 305 prokaryotic cells m^−2^ s^−1^ in the offshore samples. Moreover, the deposition flux of prokaryotes into the Red Sea is 7-fold greater than the maximum values previously reported for the global subtropical and tropical ocean. This may play a role in seeding populations that are not already present in the receiving community, thereby potentially increasing connectivity between geographically distant microbial communities (Mayol et al., 2017). Indeed, (Hu et al., 2017) report an average of 93% of airborne bacteria sampled over the Pacific Ocean to be viable, thereby potentially affecting microbial diversity. In addition to being important in regulating microbial diversity, deposition of dust-associated microbes can be functionally important. Recently (Rahav et al., 2016) reported airborne bacteria to contribute significantly to heterotrophic production following a dust deposition event in the Eastern Mediterranean, and Rahav et al. (2018) reported dust-associated diazotrophs to have an important contribution to N2-fixation in the northern Red Sea. In addition, VLPs may also affect the receiving Red Sea microbial community, as aerosolized viruses have been experimentally demonstrated that to be able to infect and kill host phytoplankton populations when re-inoculated in seawater (Sharoni et al., 2015).

## Conclusion

In conclusion, our results provide evidence of elevated loads of dust and airborne microbe over the Red Sea, particularly in summer, but no evidence for a direct relationship between loads of prokaryotes and VLPs and that of dust. Most importantly, our results demonstrate that the Red Sea is a hot spot for receiving significant loads of dust and associated airborne prokaryotes and VLPs. Furthermore, the Red Sea receives very large depositions of prokaryotes, particularly in the near-shore location, whereas airborne prokaryote and VLPs abundance are reduced to half in the Red Sea offshore locations. Deposition of airborne microbes may, therefore, be an important process affecting community dynamics and diversity of the microbial assemblages in the receiving Red Sea surface waters.

## Author Contributions

CD, MC, and JA conceived and designed the research. RY and JA conducted the research with contributions from CD and JA. RY led the data analysis and writing of the manuscript with input from all co-authors, who helped to improve the manuscript. All authors contributed to improve the manuscript and approved the submitted version.

## Conflict of Interest Statement

The authors declare that the research was conducted in the absence of any commercial or financial relationships that could be construed as a potential conflict of interest.

## Footnotes

1 https://www.ready.noaa.gov/HYSPLIT.php
