# Supplementary material for: Airborne Prokaryote and Virus Abundance Over the Red Sea
Source: Front Microbiol. 2019 May 31;10:1112. doi: 10.3389/fmicb.2019.01112 (PMC6554326; doi:10.3389/fmicb.2019.01112)

Supplementary table 1 | Cruise number, date, latitude, longitude and samples collection for TSPs, prokaryotic cells and VLPs per cruise

Supplementary Figure 1 | Sampling sites over the Red Sea. Each colored circle represents a different cruise along the red sea, while the black square represents the coastal site on the red sea at KAUST.


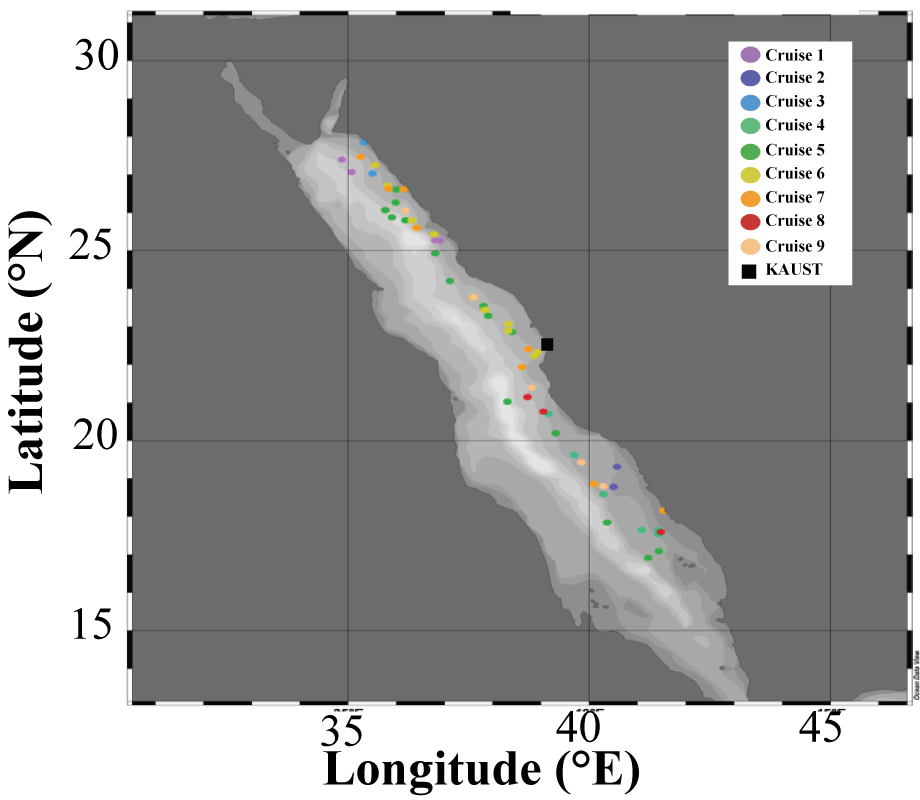

Supplement: Supplementary file 1 [file Data_Sheet_1.docx]
